# Assessing the fall risks of community-dwelling stroke survivors using the Short-form Physiological Profile Assessment (S-PPA)

**DOI:** 10.1371/journal.pone.0216769

**Published:** 2019-05-21

**Authors:** Tai-Wa Liu, Shamay S. M. Ng

**Affiliations:** 1 Department of Rehabilitation Sciences, The Hong Kong Polytechnic University, Hung Hom, Hong Kong (SAR); 2 School of Nursing & Health Studies, The Open University of Hong Kong, Ho Man Tin, Hong Kong (SAR); University of Malaya, MALAYSIA

## Abstract

**Introduction:**

Fall is common after stroke. The Short-form Physiological Profile Assessment (S-PPA) was developed to assess the fall risks and underlying physiological factors, and it has been used in healthy older adults and older adults with stroke. This study aimed to establish the psychometric properties of the S-PPA among cognitively intact and ambulant community-dwelling older adults with stroke.

**Methods:**

The S-PPA, Chinese version of the Activities-specific Balance Confidence (ABC-C) scale and 3 balance measures, namely the Berg Balance Scale (BBS), Timed “Up & Go” (TUG) and Functional Reach Test (FRT), were administered. Inter- and intra-rater reliability were assessed at baseline and after a 1-week interval, respectively. The validity of the S-PPA was assessed through correlations with balance measures and the ABC-C and by comparing the S-PPA scores between the stroke and healthy groups and between fallers and non-fallers in the stroke group. A receiver operating characteristic (ROC) curve analysis was used to assess the ability of balance measures to distinguish fall status in the stroke group.

**Results:**

The S-PPA yielded good inter-rater (intraclass correlation coefficient (ICC) = 0.83) and moderate intra-rater reliability (ICC = 0.74), correlated significantly with the 3 balance measures (rho = 0.49–0.70) and ABC-C (rho = 0.35) and revealed significant differences between stroke survivors and controls and between stroke survivors with and without a fall history. However, the ROC analysis revealed that the S-PPA had a poor ability to distinguish the fall statuses of community-dwelling stroke survivors.

**Conclusions:**

The S-PPA is reliable and valid for evaluating fall risks but cannot adequately distinguish the fall statuses of stroke survivors.

## Introduction

Stroke survivors frequently experience falls, with reported fall rates as high as 12–39% in inpatient settings and 73–80% in community settings [1]. Stroke survivors are also more susceptible than healthy individuals to serious physical injuries after falls, and particularly face a 7-fold higher risks of hip fracture [2]. Therefore, a valid, reliable, and quantitative measure of the fall risk is needed to provide insights into fall prevention and direct interventions to the underlying physiological factors of falls in stroke survivors.

Reasons of increased post stroke fall risks are multifactorial, and a recent systematic review [3] revealed that sensorimotor impairment such as balance and mobility problems, cognitive impairment, depression, self-care dependence, taking medications and history of falling are associated fall risk factors in community stroke survivors. Previous studies [4–9] have used balance measures, such as the Berg Balance Scale (BBS) [4–8] and Mini-Balance Evaluation Systems Test (Mini-BESTest) [9], and generic fall risk measures (e.g., history of previous falls) [10] to evaluate fall risks among stroke survivors. However, these balance measures have some limitations with regard to their psychometric properties and insights into fall prevention and rehabilitative interventions. For example, either a single-task balance measure, such as the Functional Reach Test (FRT) [11], or a multi-task balance measure, such as the Berg Balance Scale (BBS) [12], could be used to capture underlying physiological factors, such as vision [13] and peripheral sensation [14], that contribute to the fall risk. Although Smith and colleagues [10] tested STRATIFY [15], a generic fall risk measure, this tool was revealed to have limited ability in predicting falls (sensitivity, 11.3%; specificity, 89.5%) in stroke survivors. Furthermore, Breisinger and colleagues [16] developed a disease-specific measure of the fall risk, the Stroke Assessment of Fall Risk (SAFR), which included test items intended to assess the risk factors for falling (impulsivity and hemi-neglect) and functional balance test items (e.g., transfer) adopted from the Functional Independence Measure. Although this scale was reported to yield accurate predictions (area under the receiver operating characteristic (ROC) curve, 0.73; sensitivity, 78%; specificity, 63%) in an inpatient setting, it was unable to reveal the underlying physiological factors. Furthermore, the SAFR was not tested among community-dwelling stroke survivors and provided limited insights relevant to long-term rehabilitative planning.

Lord and colleagues [14] developed the Physiological Profile Approach (PPA) to assess the fall risks of older adults. The main advantage of this approach is the conceptualization of the fall risk as an interaction of multiple sensorimotor factors, rather than only the individual’s balance performance, the presence of demographic risk factors (e.g., age) or clinical characteristics (e.g., use of medication). Rather, the PPA assesses 5 physiological factors, including vestibular function, peripheral sensation, muscle force, vision and reaction time, contributing to the balance and mobility performance which are associated with the fall risk factors common to both the older adults and stroke survivors (3, 14).

Long and short forms of the PPA are available. The long form includes 13 individual test items, including 3 vision tests (high- and low-contrast visual acuity, contrast sensitivity and visual field dependence), 3 peripheral sensation tests (tactile sensitivity, vibration sense and proprioception), 3 muscle force tests (knee flexion, knee extension and ankle dorsiflexion), 2 reaction time tests (hand and foot) and 2 postural sway tests (standing on the floor and standing on a foam rubber mat with or without eyes closed). The long form requires a total of 45 minutes to complete. However, the short form (S-PPA), which retains 5 of the 13 individual item tests on the long-form PPA, was developed to reduce the administrative time for screening purposes [14]. These 5 retained individual test items, namely the Melbourne Edge Test, proprioception test, knee extension muscle strength test, hand reaction time test and postural sway test, exhibited moderate to excellent test-retest reliability (r = 0.50–0.97) in community-dwelling older people [14]. These 5 tests could also correctly distinguish 75–79% of older adults with or without a fall history in institutional [17] and community settings [18].

The assessment of fall risks and the underlying physiological factors could facilitate fall prevention strategies and rehabilitative training for stroke patients. Although the PPA has been applied to populations of older adults [17, 18], including those with various chronic illnesses such as stroke [19, 20], Parkinson disease [21] and multiple sclerosis [22], the psychometric properties of the PPA have not been systematically investigated in stroke survivors. Therefore, the objectives of this study were to investigate the inter-rater and intra-rater reliability, concurrent, convergent and known-group validity and accuracy of the S-PPA for distinguishing the fall history in chronic stroke survivors.

## Materials and methods

### Subjects

One hundred and thirty-seven individuals (70 men, 67 women) with a history of single stroke within 1–6 years, as confirmed by magnetic resonance imaging or computed tomography, were recruited from a local support group through poster advertisements. The inclusion criteria were an age between 50 and 85 years, ability to walk independently for at least 10 meters with or without an assistive device and a score of ≥7/10 on the Chinese version of the Abbreviated Mental Test (AMT-C) [23]. The exclusion criteria were as follows: any additional medical condition (e.g., angina pectoris), visual impairment not correctable by glasses, musculoskeletal pain during daily activities or significant lower limb impairment that could affect the assessment (e.g., foot drop).

Forty age-matched healthy participants (18 men, 22 women) were recruited at Hong Kong Polytechnic University through poster advertisements. Individuals with any neurological condition (e.g., Parkinson’s disease) and an AMT-C score <7 were excluded.

Ethical Approval has been granted by Human Subjects Ethics Committee of The Hong Kong Polytechnic University (HSEARS20131012002-01). This study was conducted according to the principles of the Declaration of Helsinki regarding human experiments. Informed consent was obtained from the participants prior to the experiment.

### Outcome measures

#### Short-form Physiological Profile Assessment (S-PPA)

The S-PPA is a physiological-oriented measure of the fall risk consisting of 5 sensorimotor and balance performance items:

1. The Melbourne Edge Test was used to assess visual contrast sensitivity. Subjects were required to identify the directions of the low- and high-contrast edges of 20 circular patches (diameter, 25 mm) on a chart. The directions included horizontal, vertical and 45° left and right. The subjects were provided an instructional key card containing the 4 possible directions of the contrasting edges. The correct identification of the lowest contrasting edge was recorded in decibel units (dB). This test yielded good test-retest reliability (intraclass correlation coefficient (ICC) = 0.81, 95% CI: 0.70–0.88) in community-dwelling older adults[14].
2. The proprioception test was used to assess proprioception. The seated subjects were asked to align their great toes simultaneously on either side of a vertical acrylic-plastic plate (60 cm x 60 cm x 1 cm) without looking at the plate. Any differences in the alignment of the great toes were recorded in degrees. This test yielded moderate test-retest reliability (ICC = 0.50, 95% CI: 0.15–0.74) [14].
3. The hand reaction time test was used to assess the response time. Subjects were required to press the response switch of a modified computer mouse when a red light adjacent to the switch was activated. The reaction time was measured in milliseconds using a built-in timer. This test yielded moderate test-retest reliability (ICC = 0.69, 95% CI: 0.45–0.84) [14].
4. The knee extension strength test was used to evaluate the strength of the quadriceps muscle in the affected leg. Subjects were required to remain seated while a strap connected to a spring gauge was placed around the leg. The knee extension strength was measured in kilograms using the spring gauge. This test yielded excellent test-retest reliability (ICC = 0.97, 95% CI: 0.93–0.98) [14].
5. The postural sway test was used to assess body displacement. A belt was tied to the subject’s waist for 30 seconds. A rod (length, 40 cm) was connected to the belt, and a pen attached to the end of the rod was used to record the subject’s body movement on graph paper in mm^2^. This test yielded moderate test-retest reliability (ICC = 0.68, 95% CI: 0.45–0.82) [14].

The results of each individual item test were input into a Web-based computer software program and converted into individual standardized (z) scores and composite score. Using reference data from previous studies [17, 18], the composite scores were further converted into standardized (z) 7-point composite fall risk scores of -2 to 4, in which scores of -2 to 0, 0 to 2 and 2 to 4 indicate a very low to low, low to marked and marked to very marked fall risk, respectively. The item tests were administered according to standardized instructions.

#### Berg Balance Scale (BBS)

The BBS is a 14-item measure used to evaluate functional balance on a 5-point scale. A higher composite score indicates a better balance performance. In stroke survivors, this measure yielded good to excellent test-retest reliability (ICC = 0.88–0.92) [24, 25] and strong concurrent validity with the Postural Assessment Scale for Stroke Patients (r = 0.92–0.95) [26].

#### Functional Reach Test (FRT)

The FRT was used to evaluate standing balance by measuring the maximum distance the subject could reach forward while standing on a fixed base. In stroke survivors, this test yielded excellent test-retest reliability (ICC = 0.99) [27] and moderate to strong concurrent validity (r = 0.62–0.78) with the BBS [8, 27].

#### Timed “Up & Go” (TUG)

The TUG [28] was used to evaluate functional mobility by measuring the time required by subjects to perform sequential motor tasks, including rising from an armchair, walking 3 m, turning and walking back and sitting down again. In stroke survivors, this measure yielded good to excellent test-retest reliability (r = 0.87–0.99) [29, 30] and excellent concurrent validity (r = 0.99) [30] with gait velocity.

#### The Chinese version of the activities-specific balance confidence scale (ABC-C)

The ABC-C [31] is a 16-item measure used to assess a subject’s subjective balance confidence (0%, no confidence; 100%, complete confidence) in specific daily life situations, such as “getting into or out of a commonly used form of transportation”. In stroke survivors, the ABC yielded good test-retest reliability (ICC = 0.85) and moderate concurrent validity with the TUG (r = 0.48), 10-meter walk test (10MWT) (r = 0.52) and 6-minute walk test (r = 0.45) [32].

### Data collection

The assessments were performed in a university-affiliated neurorehabilitation laboratory. After providing written consent, the participants completed a data extraction form, including demographic data, history of falls in the past 12 months, types of medications and co-morbid conditions, and the ABC-C. Subsequently, the participants completed the balance tests in random order. These tests were conducted by a physiotherapist with >5 years of clinical experience (rater 1).

### Experimental protocol

A resting interval of ≥5 minutes was allowed between each balance test and each S-PPA individual item test. Additional resting time was allowed if needed. The measurements were conducted according to the following procedure:

1. TUG: subjects were allowed 1 practice trial and then completed 3 test trials. The average time of the 3 test trials was used.
2. FRT: subjects were allowed 2 practice trials and then completed 3 test trials. The average distance of the 3 test trials was used.
3. BBS: subjects were asked to complete 1 BBS trial. Instructions were provided by rater 1.
4. S-PPA: subjects were asked to complete 1 trial of the Melbourne Edge Test and 1 trial of the postural sway test. Instructions were provided by rater 1. The subjects were allowed 1 practice trial for the proprioception, and 5 pre-practice tests and 5 practice for the hand reaction time tests, respectively, after instructions were provided by rater 1. Next, the participants were asked to complete 5 trials of the proprioception test and 10 trials of the hand reaction time test, respectively. For the knee extension strength test, subjects were asked to complete 3 trials after instructions were provided by rater 1, and the average weight lifted in the 3 trials was calculated.

Twenty-eight consecutive participants were included in the assessment of inter-rater reliability between 2 physiotherapists (raters 1 and 2). These assessments were conducted simultaneously at baseline, with no discussion or disclosure of scores. To determine intra-rater reliability, another 28 consecutive participants not involved in the assessment of inter-rater reliability were re-assessed according to the S-PPA experimental protocol after a 1-week interval. One of the same physiotherapists (rater 1) conducted this assessment at the same time as the first assessment. The healthy controls completed the demographic data sheet and underwent the S-PPA once as conducted by rater 1.

### Data analysis

The collected quantitative data were analyzed using SPSS 20.0 software at a significance level of α = 0.05. Descriptive statistics were used to summarize the demographic data and variables of interest. The normality of data and homogeneity of the variances were checked using the Shapiro–Wilk test and Levene’s test, respectively.

ICCs were used to establish test-retest reliability, and the equation used to determine this value was selected depending on the intent of the analysis [33]. We adopted the ICC (2, 1) to determine inter-rater reliability and generalize the results of our findings to those of other S-PPA raters for clinical practice or research trials. We adopted the ICC (3, 1) to determine intra-rater reliability between the 2 specified raters (raters 1 & 2) [33]. ICCs of >0.90, 0.75–0.90, 0.50–0.75 and <0.50 indicate excellent, good, moderate and poor reliability, respectively [34].

To evaluate concurrent validity, the correlations of the S-PPA scores with the, FRT, TUG and BBS scores were examined. Convergent validity was examined using the correlation between the test S-PPA and ABC-C scores. Pearson’s r and Spearman’s rho analyses were used to analyze the correlations between normally and non-normally distributed variables, respectively. To compare fallers with non-fallers in the stroke group and the stroke group with the healthy group and assess known-group validity, independent t-tests were applied to parametric data and the Mann–Whitney U test was applied to non-parametric data. Fallers were identified as subjects who had at least one falls in the last 12-month period before data collection. Non-fallers were those with no fall experiences in the 12-month period.

A receiver operating characteristic (ROC) curve analysis and areas under the curve (AUCs) were used to determine the cutoff point of the S-PPA composite score. AUC values of ≤0.5, 0.5 to <0.7, 0.7 to <0.8, 0.8 to <0.9 and ≥0.9 indicate no, poor, acceptable, excellent and outstanding discrimination, respectively [35]. The cutoff score was determined using the Youden index at the point where both the sensitivity and specificity values were maximized.

## Results

### Characteristics of the subjects

The stroke group had a mean age of 61.2 years, and a mean interval of 3.1 years since the stroke event (Table 1). Seventy-eight (57%) of the patients had 2 or more other chronic comorbidities, such as hypertension, and most of them (n = 109, 80%) were taking 2 or more medications (e.g., hypertensive agents). Eighty-three of the patients (61%) had right-side hemiplegia, and the majority (n = 129, 94%) required walking aids when walking outdoors. The majority of the patients (n = 96, 70%) had no history of fall during the 12 months before the study began.

**Table 1:** Demographics of participants.

	Stroke group (N = 137) Value, mean+SD	Healthy group (N = 40) Value, mean+SD	t or χ^2^ (*p*-value)
Age (y)	61.2±7.2	62.4 ± 5.1	-0.95 (0.342)
Sex, n (%)			0.36 (0.339)
Female	68 (50)	22 (55)	
Male	69 (50)	18 (45)	
BMI (kg/m^2^)	24.3±3.4	23.3 ± 2.7	1.92 (0.058)
Living arrangement., n (%)			
Alone	11 (8)		
With family/carer	126 (92)		
Education., n (%)			
Primary or below	30 (22)		
Secondary	86 (63)		
University or college	21 (15)		
Years since stroke	3.1± 1.7		
Cause of stroke, n (%)			
Ischemic	56 (41)		
Hemorrhagic	77 (56)		
Unknown or mixed	4 (3)		
Hemiplegic side, number			
Left/right	54/83		
Number of chronic medical conditions, n (%)			
0	20 (15)		
1	39 (29)		
2	56 (41)		
3 or above	22 (15)		
Number of medication, n (%)			
0–1	28 (20)		
2–3	65 (47)		
4 or above	44 (33)		
History of falls in past 12 months, n (%)			
0	96 (70)		
1	35 (26)		
2 or above	6 (4)		
Mobility status, number			
Unaided	8 (6)		
Stick	106 (77)		
SBQ	15 (11)		
LBQ	8 (6)		

Note: SD, standard deviation; n, number, BMI, body mass index; SBQ, small base quadripod; LBQ, large base quadripod; t, t test; χ^2^, chi-square.

Table 2 summarizes the individual test items and S-PPA composite scores of the stroke and healthy groups. The mean S-PPA, FRT, TUG, BBS and ABC-C scores of the stroke group were 1.2, 20.3, 17.2, 49.2 and 65.1, respectively. As expected, the healthy group demonstrated better performance on the S-PPA when compared to the stroke group, as demonstrated by the mean composite and individual test item scores.

**Table 2:** S-PPA, FRT, TUG, BBS and ABC-C, TUG and of the stroke group and healthy group.

	Stroke group (N = 137)	Healthy group (N = 40)
S-PPA composite score (lower better)	1.2±0.9	0.2± 0.8
Melbourne Edge Test (dB) (higher better)	19.8±1.9	20.2±2.1
Proprioception test (degrees) (lower better)	6.8±3.3	2.0±1.0
Hand reaction time test (ms) (lower better)	283.1±58.3	245.2±42.0
Knee extension strength test (kg) (higher better)	34.1±11.6	49.8±12.3
Postural sway test (mm^2^) (lower better)	781.4±665.3	544.7±363.6
FRT (cm) (higher better)	20.3±3.0	
TUG (s)(lower better)	17.2±5.0	
BBS (lower better)	49.2±3.8	
ABC-C (lower better)	65.1±18.4	

Note: values are mean ± SD. FRT, Functional reach test; TUG, Timed “Up & Go”; BBS, Berg Balance Scale; ABC-C, Chinese version of the activities-specific scale; S-PPA, Short–form Physiological Profile Assessment.

### Reliability

The ICCs for the inter-rater and intra-rater reliability of the S-PPA composite score were 0.83 (95% CI: 0.67–0.92, p<0.001) and 0.74 (0.51–0.87, p<0.001), respectively (Table 3). The ICCs for the inter-rater reliability of the S-PPA individual items ranged from 0.56 (95% CI: 0.24–0.77, p = 0.001) to 0.87 (95% CI: 0.74–0.94, p<0.001), and those for the intra-rater reliability ranged from 0.58 (95% CI: 0.28–0.78, p<0.001) to 0.94 (95% CI: 0.87–0.97, p<0.001).

**Table 3:** Intra-rater and interrater reliability statistics for S-PPA.

S-PPA section	Interrater reliability		Intra-rater reliability	
	ICC, Mean (95% CI)	p-value	ICC, Mean (95% CI)	p-value
Melbourne Edge Test	0.87 (0.74–0.94)	<0.001	0.89 (0.78–0.95)	<0.001
Proprioception test	0.60 (0.30–0.80)	<0.001	0.62 (0.32–0.80)	<0.001
Knee extension strength test	0.86 (0.72–0.93)	<0.001	0.94 (0.87–0.97)	<0.001
Hand reaction time test	0.83 (0.67–0.92)	<0.001	0.89 (0.77–0.95)	<0.001
Postural sway test	0.56 (0.24–0.77)	0.001	0.58 (0.28–0.78)	<0.001
S-PPA composite score	0.83 (0.67–0.92)	<0.001	0.74 (0.51–0.87)	<0.001

Note: CI, confidence interval; S-PPA, Short–form Physiological Profile Assessment.

### Concurrent and convergent validity

Significant correlations were observed between the S-PPA composite score and various balance measures, including the BBS (rho = -0.70, p<0.001), FRT (rho = -0.57, p<0.001) and TUG (rho = 0.49, p<0.001). Furthermore, a significant negative correlation was observed between the S-PPA composite score and ABC-C score (rho = -0.35, p<0.001).

### Known-group validity

A comparison of fallers and non-fallers in the stroke group revealed significant differences in the S-PPA composite score (*U* = 1,495.5, p = 0.026) and in the vision test (*U* = 1,489.5, p = 0.022) (Table 4). A comparison of the stroke and healthy control groups revealed significant differences in the S-PPA composite score (*U* = 1,166.0, p<0.001) and most of the individual item scores, including the proprioception test (*U* = 551.5, p<0.001), knee extension strength test (*U* = 988.5, p<0.001), hand reaction time test (*U* = 1,532.0, p<0.001) and postural sway test (*U* = 2,100.0, p = 0.03).

**Table 4:** Known-group validity of the S-PPA.

	Stroke group (N = 137)	Healthy group (N = 40)	Stroke group			
	Mean rank	Mean rank	p-value	Fallers (N = 41)	Nonfallers (N = 96)	p-value
				Mean rank	Mean rank	
Melbourne Edge Test (dB)	86.43	97.81	0.209	57.33	73.98	0.022
Proprioception test (degrees)	104.97	34.29	<0.001	74.73	66.55	0.269
Knee extension strength test (kg)	76.22	132.79	<0.001	66.37	70.13	0.612
Hand reaction time test (ms)	97.82	58.80	<0.001	75.43	66.26	0.216
Postural sway test (mm^2^)	93.67	73.00	0.025	78.35	65.01	0.071
S-PPA composite score	100.49	49.65	<0.001	80.51	64.08	0.026

Note: S-PPA, Short–form Physiological Profile Assessment; n, number.

### ROC curve analysis

**Fig 1:**
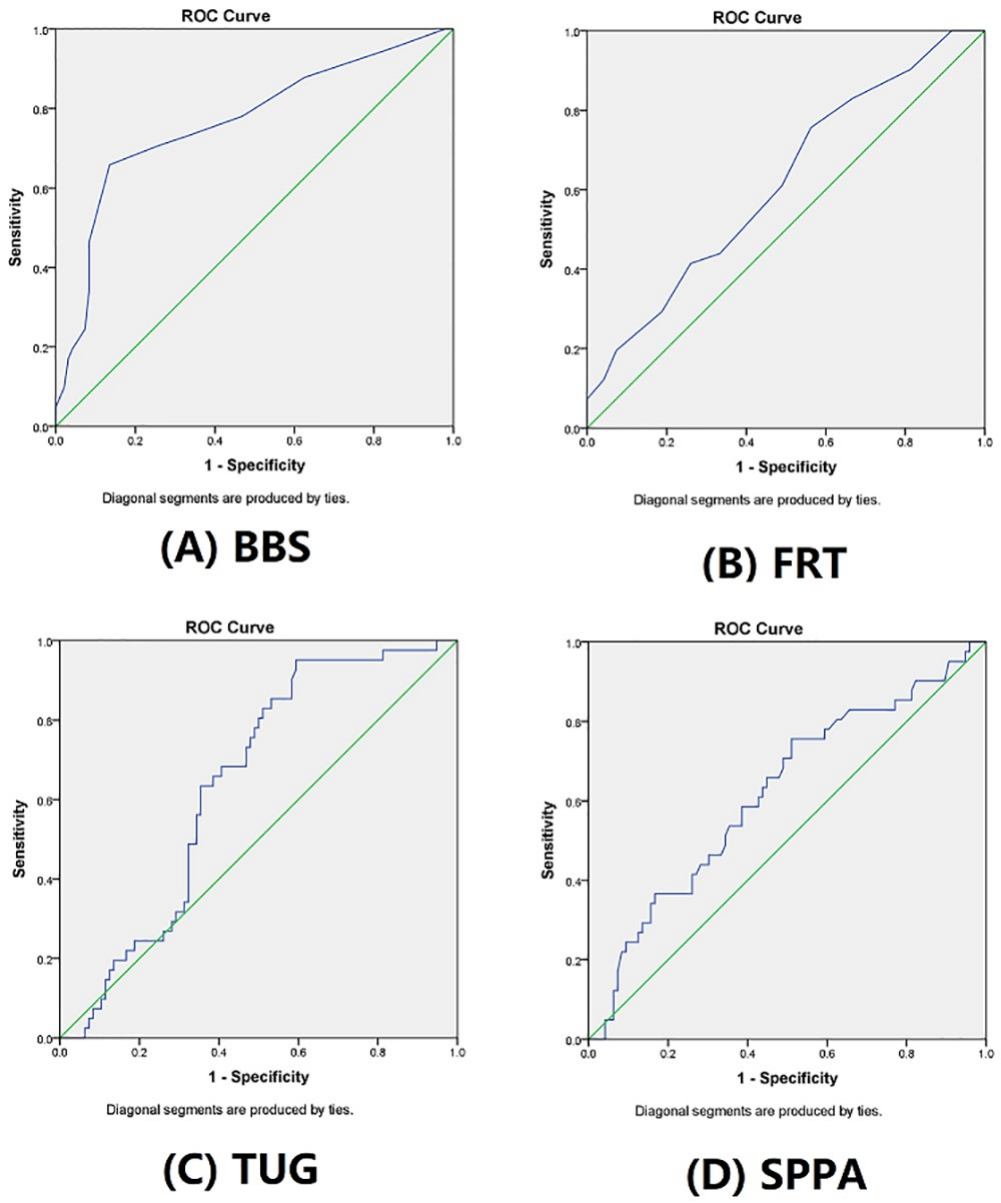
Receiver operator characteristics of the (A) Berg Balance Scale (BBS), (B) Functional Reach Test (FRT), (C) Timed Up and Go (TUG) and (D) Short-form Physiological Profile Assessment (S-PPA) to distinguish stroke subjects with or without a history of fall (N = 137).

Table 5 summarizes the results of the ROC curve analysis of the fall risk screening tests. A S-PPA cutoff score of 0.87 was identified between fallers and non-fallers in the stroke group, and the ROC curve yielded an AUC of 0.62 (95% CI: 0.52–0.72), sensitivity of 39% and specificity of 81%. Among the 4 tests used to screen the fall risk, only the BBS demonstrated an acceptable ability to differentiate participants with stroke who did or did not have a history of falls (AUC = 0.77, 95% CI: 0.67–0.86). The remaining 3 screening tests of fall risk, namely the S-PPA, FRT and TUG, had a poor discriminative ability to distinguish participants with stroke who did or did not have a history of falls (AUC = 0.62–0.64). The AUCs of the balance measures are illustrated in Fig 1.

**Table 5:** Ability of BBS, FRT, S-PPA and TUG to distinguish stroke survivors with or without fall history.

Fall risk screening tests	AUC	95%CI	Cutoff point	Sensitivity % (95% CI)	Specificity % (95% CI)
BBS	0.77	0.67–0.86	47.5	68 (52–80)	86 (77–91)
FRT	0.62	0.52–0.72	21.5	36 (27–47)	81 (68–89)
S-PPA	0.62	0.52–0.72	0.87	39 (29–50)	82 (71–90)
TUG	0.64	0.55–0.73	14.21	41 (31–51)	95 (84–99)

Note: AUC, area under the characteristic; CI, confidence interval; BBS, Berg Balance Scale; FRT, Functional reach Test; S-PPA, Short–form Physiological Profile Assessment; TUG, Timed “Up & Go”.

## Discussion

This was the first study to psychometrically test the S-PPA using stroke survivors and age-matched healthy controls. All of the participants were able to complete the S-PPA, indicating that this fall risk measure could be feasibly applied to stroke survivors with intact cognition (AMT score ≥7) and mild balance impairments. Our findings revealed that the both S-PPA composite score and individual test scores yielded moderate to good interrater and intra-rater reliability in this cohort of ambulant community-dwelling stroke survivors. The S-PPA was found to exhibit significant moderate correlations with measures of balance, including the BBS, FRT and TUG, and the ABC-C. Our finding also demonstrated that the S-PPA can distinguish the fall risks of community-dwelling chronic stroke survivors and of healthy older adults. However, the S-PPA composite score had a limited ability to discriminate between fallers and non-fallers among the included stroke survivors.

Our findings regarding test-retest reliability are consistent with those reported by Lord and colleagues [14, 18], who tested samples of community-dwelling older adults. Our findings and those reported by Lord and colleagues [14, 18] revealed that the knee extension strength test had excellent test-retest reliability (ICC = 0.97, 95% CI: 0.93–0.98) [14], the Melbourne Edge test had good test-retest reliability (ICC = 0.81, 95% CI: 0.70–0.88) and the proprioception test (ICC = 0.50, 95% CI: 0.15–0.74) [14] and postural sway test (ICC = 0.68, 95% CI: 0.45–0.84) [18] had moderate test-retest reliability. However, our analysis of the test-retest reliability of the proprioception test (inter-rater reliability, ICC = 0.60, 95% CI: 0.30–0.80; intra-rater reliability, ICC = 0.62, 95% CI: 0.32–0.80) yielded better results than those obtained by Lord and colleagues [14] (ICC = 0.50, 95% CI: 0.15–0.74). One possible explanation for this discrepancy is that our study participants were relatively younger (mean age: 61±7 years) than those recruited by Lord and colleagues (mean age: 81±3 years) [14], and the relatively better peripheral sensation of the younger participants may have yielded more stable scores during repeats of the proprioception test. Indeed, in a study of 550 community-dwelling women aged 20–99 years, Lord and colleagues [18] revealed that peripheral sensation, as measured by proprioception, correlated significantly with age (r = 0.20, p<0.0001). Furthermore, the test-retest reliability of the postural sway test was poorer in this study (ICC = 0.56–0.58) than the value reported by Lord and colleagues [14] (ICC = 0.68), consistent with previous studies in which the test-retest reliability of postural sway was comparatively better in healthy adults (test-retest reliability ICC = 0.33–0.93) [36–38] than in populations of patients with such as chronic low back pain (ICC = 0.26) [36] and transtibial amputation (ICC = 0.67) [39]. Possibly, a post-stroke asymmetric weight-bearing capacity could lead to an increase in postural sway [40], which might thus affected the stability of postural control during repeats of the postural sway test. Our findings also revealed that the S-PPA composite score has a higher degree of inter-rater reliability (ICC = 0.83) than intra-rater reliability (IC = 0.74). One possible explanation is that there may be unspecified measurement error between raters such as the height of the adjustable table used for recording the subject’s body movement during postural sway test. Of course, despite the test-retest interval of only one week, we cannot completely exclude the possibility that the study participants’ physiological factors were improved or deteriorated.

We found that among stroke survivors in this study, the S-PPA composite score correlated significantly with the balance measures of FRT, TUG and BBS (rho = -0.49 to 0.70), but exhibited a comparatively weaker correlation with the ABC-C (rho = -0.35). This finding is inconsistent with previous findings that the subjective balance confidence, as measured by the ABC, correlates with several balance measures, including the TUG (r = -0.48), 10MWT (r = -0.52) and 6-minute walk test (r = 0.45) [32]. We believe that this discrepancy can be attributed to the different aspects of balance measured by these instruments. Whereas the TUG, 10WMT and 6-minute walk test are measures of the functional balance performance, the S-PPA measures aspects of the physiological profile that contribute to the fall risk, including vision, muscle force, peripheral sensation, reaction time and postural sway. The items on the ABC-C are directly related to daily activities (e.g., getting into or out of frequently used transportation) and functional balance (e.g., distance walking, changing directions and transit postures), as community-dwelling stroke survivors require confidence to perform these activities and functional balance is expected to correlate with the subjective balance confidence. However, the adverse effects of physiological deficits on functional balance could be compensated by external factors, such as the use of walking aids or walking with a companion. In summary, this study identified a limited association between the S-PPA and ABC-C.

The known-group validity of the S-PPA was demonstrated by its ability to distinguish people with and without stroke and those with and without a fall history among stroke survivors. Our findings are consistent with the findings of Lorbach and colleagues [41], who reported that the S-PPA composite score can effectively distinguish between people with and without Alzheimer’s disease. As expected, the performances of stroke survivors were worse than those of healthy controls in most of the S-PPA individual item tests, except the Melbourne Edge Test. Among stroke survivors, we found that those with a history of fall within a 1-year period before the study began had a poorer S-PPA composite score but did not receive worse scores on any individual item test. These findings might suggest that for community-dwelling stroke survivors with intact cognition, the adoption of fall prevention strategies such as the avoidance of high-risk situations could effectively compensate for the physiological limitations associated with aging and chronic illness. In other words, the S-PPA did not identify notable differences among the physiological deficits in this study.

Our ROC curve analysis revealed findings consistent with those reported by Tsang and colleague [9], who stated that the BBS was best able to distinguish stroke survivors who did and did not have a history of falls (AUC = 0.72, 95% CI: 0.61–0.83) when compared with the FRT and TUG. In contrast to previous prospective studies demonstrating the good predictive ability of the PPA among older adults with or without a fall history, our retrospective findings suggested that the S-PPA had a poor ability to distinguish stroke survivors with and without a fall history. The inconsistencies between the studies by Lord and colleagues [17, 18] and our findings may be explained by differences in the study population; the former included older adults who resided in an intermediate care institution and hostel for aged persons. Although the participants generally exhibited independence in activities of daily living, their participation in social activities may have been comparatively limited and their living environments might have been altered to reduce fall hazards. By contrast, our study participants were community-dwelling and participated actively in exercise and social activities. Therefore, the falls experienced by our study participants were more likely attributable to external risk factors, compared to those of older adults who resided in more structured living environments.

As reviewed by Xu and colleagues [3], sensorimotor performance is one of the fall risk factors of community-dwelling people with stroke. Together with Xu and colleagues [3] and our findings, we inferred that the S-PPA could reliably assess one of the risk factors leading to increased fall risks, namely the impacts of physiological factors on sensorimotor performance. However, S-PPA’s ability to screen for overall fall risks of community-dwelling people with stroke is limited.

This study had several major limitations. First, the generalization of our findings is limited to other populations that meet our inclusion and exclusion criteria. Second, this study had a cross-sectional design, and the ability of the S-PPA to identify fallers was analyzed retrospectively. Finally, men might have been underrepresented in our sample of stroke survivors as previous evidence revealed that men had higher preponderance rate of stroke survivors across age groups in some countries [42].

## Conclusions

The S-PPA is a valid and reliable measure of the fall risks faced by chronic stroke survivors. Although this measure demonstrated a limited ability to discriminate between stroke survivors with and without a history of falling, it was found to exhibit moderate concurrent validity with several balance measures, including the BBS, FRT and TUG, and moderate convergent validity with the ABC-C. The S-PPA is advantageous because it used simple equipment to assess the physiological factors that might contribute to the fall risks of stroke survivors. Therefore, this measure provides useful information that can be used to personalize physiological treatment regiments to meet the needs of for stroke survivors with balance disorders. Further studies of patients with a variety of stroke-specific impairments present at several levels of severity would provide additional data for validation and generalizability.

## Supporting information

S1 File This is the S-PPA dataset. (XLS)
